# Isolated Persistent Left Superior Vena Cava Causing Hemodialysis Catheter Dysfunction: An Uncommon Challenge in Vascular Access

**DOI:** 10.7759/cureus.97013

**Published:** 2025-11-16

**Authors:** Julian Müller-Kühnle, Chiara Stäbler, Sarah Mandel, Johanna Sauer, Matthias Gruber, Alexander Maßmann, Severin Schricker, Inga Hammer, Jörg Latus, Moritz Schanz

**Affiliations:** 1 General Internal Medicine and Nephrology, Robert Bosch Hospital, Stuttgart, DEU; 2 Radiology and Nuclear Medicine, Robert Bosch Hospital, Stuttgart, DEU

**Keywords:** central venous catheter (cvc), computed tomography angiography (cta), hemodialysis access, persistent left superior vena cava (plsvc), radiologic imaging

## Abstract

A persistent left superior vena cava (PLSVC) is the most common congenital anomaly of the thoracic venous system. Complete absence of the right superior vena cava (SVC) is rare but may complicate central venous catheter placement and hemodialysis access if unrecognized. We report the case of a 76-year-old man with dilated cardiomyopathy, heart failure with reduced ejection fraction, and end-stage kidney disease who was admitted with volume overload and metabolic acidosis requiring urgent hemodialysis. A tunneled central venous catheter inserted via the right internal jugular vein showed an atypical leftward guidewire course on fluoroscopy, and digital subtraction angiography revealed a PLSVC. Computed tomography angiography confirmed a large-caliber PLSVC draining into a markedly dilated coronary sinus with complete absence of a right-sided SVC. Based on these findings, vascular access was re-established via the left internal jugular vein with the catheter positioned deeper to ensure correct placement, which restored effective dialysis flow. This case underscores that an isolated PLSVC, although uncommon, can significantly affect vascular access procedures. Early recognition of an atypical guidewire trajectory and prompt imaging are essential for safe and functional hemodialysis catheter placement.

## Introduction

A persistent left superior vena cava (PLSVC) is the most common congenital venous anomaly of the thorax, occurring in approximately 0.1-0.5% of the general population and in up to 4% of individuals with structural heart disease [1-3]. It results from incomplete regression of the left anterior cardinal vein during embryogenesis, leading to persistence of a left-sided caval channel [4]. In most cases, a right superior vena cava (SVC) coexists, and the variant remains asymptomatic [5,6]. In contrast, the absence of the right SVC occurs in fewer than 0.1% of cases, where all venous return from the head, neck, and upper extremities drains through the PLSVC into the coronary sinus [7,8].

Although a PLSVC is typically asymptomatic, its presence can have important procedural implications. It may complicate central venous access - particularly during central venous catheter placement, cardiac device implantation, or the establishment of hemodialysis access - by increasing the likelihood of guidewire deviation, catheter malposition, and suboptimal flow compared with normal venous anatomy [9-11]. If the anomaly remains unrecognized during catheter placement, the altered venous course can lead to unexpected wire movement, difficulty achieving optimal catheter positioning, or reduced flow efficiency during subsequent use. Early recognition - through fluoroscopy or cross-sectional imaging - is essential to prevent such complications and to ensure effective vascular access.

We report a rare case of an isolated persistent left superior vena cava, identified during the insertion of a tunneled hemodialysis catheter, which resulted in catheter dysfunction and required contralateral reimplantation. To our knowledge, such presentations are infrequently described in the setting of tunneled dialysis access, underscoring practical imaging clues and access-strategy considerations that may help avoid ineffective catheter function without introducing additional risk.

## Case presentation

A 76-year-old man with a history of dilated cardiomyopathy, chronic heart failure with reduced ejection fraction (HFrEF), paroxysmal atrial fibrillation (CHA₂DS₂-VASc score 5, on oral anticoagulation with rivaroxaban), and chronic kidney disease stage G5 secondary to hypertensive and diabetic nephropathy, was referred to our department for initiation of chronic hemodialysis.

The patient presented with progressive dyspnea at rest, massive bilateral leg edema, ascites, and anasarca despite high-dose torasemide (80 mg daily). On examination, he appeared in reduced general condition with a hypotensive blood pressure of 90/40 mmHg and clinical signs of right heart failure, including distended jugular veins and hepatomegaly.

Initial laboratory investigations demonstrated severe renal dysfunction with marked azotemia and metabolic acidosis, consistent with uremic decompensation in the context of end-stage kidney disease (Table 1).

**Table 1:** Baseline laboratory findings on admission. Initial laboratory evaluation revealed severe azotemia, metabolic acidosis, and markedly elevated NT-proBNP, consistent with uremic decompensation in the setting of end-stage kidney disease. Reference intervals represent institutional standard values. NT-proBNP, N-terminal pro-B-type natriuretic peptide; eGFR, estimated glomerular filtration rate; CKD-EPI, Chronic Kidney Disease Epidemiology Collaboration (eGFR equation)

Parameter	Result	Reference range	Interpretation
White blood cells	6.1 × 10⁹/L	3.9-10.2 × 10⁹/L	Normal
Hemoglobin	12.7 g/dL	13.5-17.5 g/dL	Mildly decreased
Platelets	155 × 10⁹/L	150-400 × 10⁹/L	Normal
Ionized calcium	1.12 mmol/L	1.15-1.30 mmol/L	Slightly decreased
C-reactive protein (CRP)	0.9 mg/dL	< 0.5 mg/dL	Mildly elevated
Sodium	141 mmol/L	135-145 mmol/L	Normal
Potassium	4.0 mmol/L	3.5-5.0 mmol/L	Normal
Chloride	103 mmol/L	98-107 mmol/L	Normal
Bicarbonate	14 mmol/L	22-29 mmol/L	Decreased (metabolic acidosis)
NT-proBNP	30998 pg/ml	<879 pg/mL	Markedly elevated
Urea	94 mg/dL	17-43 mg/dL	Markedly elevated
Creatinine	3.7 mg/dL	0.7-1.2 mg/dL	Markedly elevated
eGFR (CKD-EPI)	15 mL/min/1.73 m²	> 60 mL/min/1.73 m²	Severely decreased
Prothrombin time (Quick)	57 %	70-130 %	Prolonged
INR	1.37	0.8-1.2	Mildly elevated

**Figure 1:**
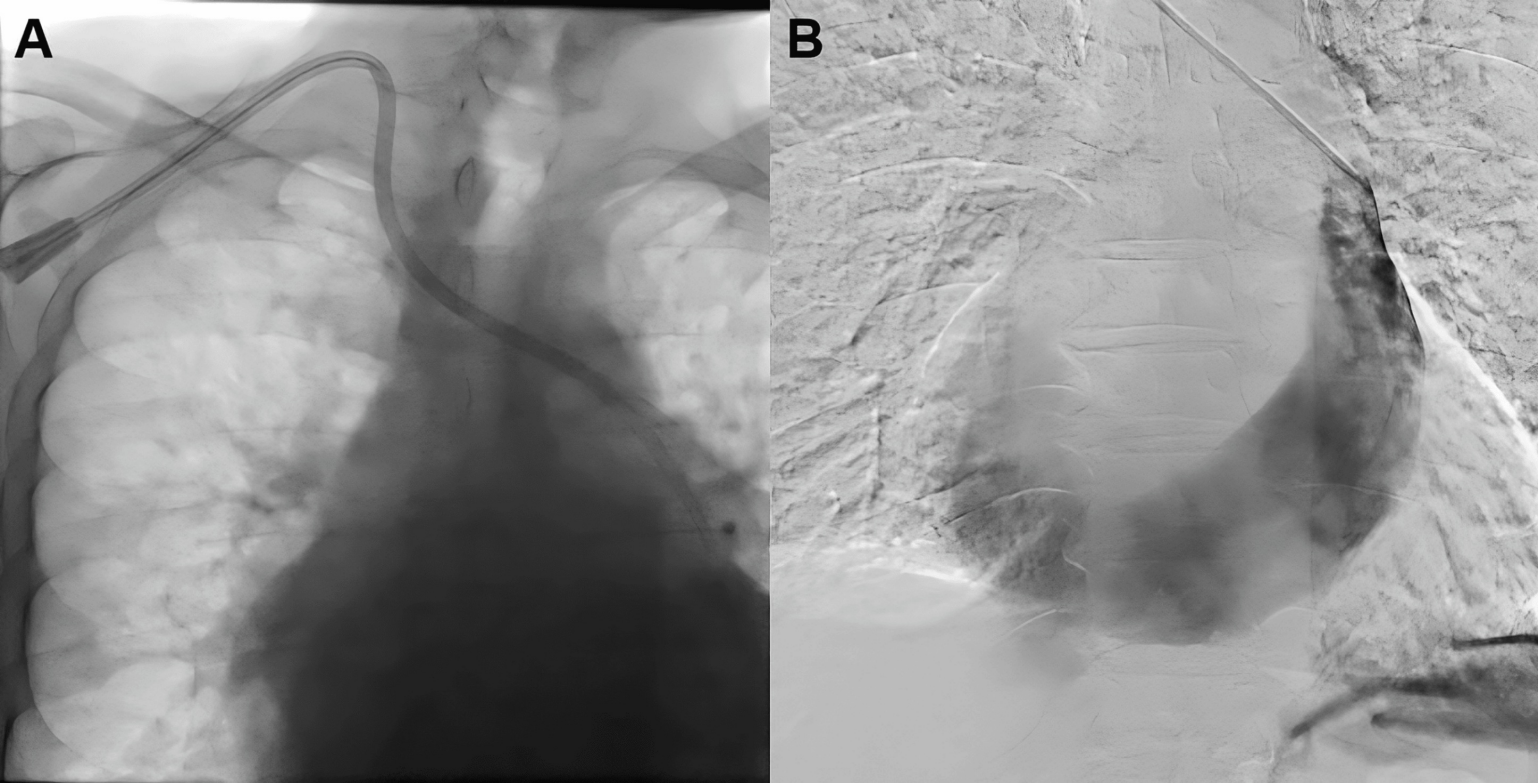
Fluoroscopic and angiographic confirmation of a persistent left superior vena cava (PLSVC) during right internal jugular catheterization. (A) Fluoroscopic image during right internal jugular venous catheterization demonstrating an atypical leftward mediastinal course of the guidewire and catheter, suggesting an anomalous venous pathway. (B) Digital subtraction angiography (DSA) following contrast injection through the right internal jugular sheath, confirming opacification of a PLSVC draining into the coronary sinus.

On October 14, 2025, a tunneled double-lumen central venous dialysis catheter was placed via the right internal jugular vein under ultrasound and fluoroscopic guidance for urgent dialysis initiation. After venipuncture, the guidewire demonstrated an atypical leftward mediastinal course on digital subtraction angiography (DSA). Contrast injection revealed a PLSVC (Figure 1).

**Figure 2:**
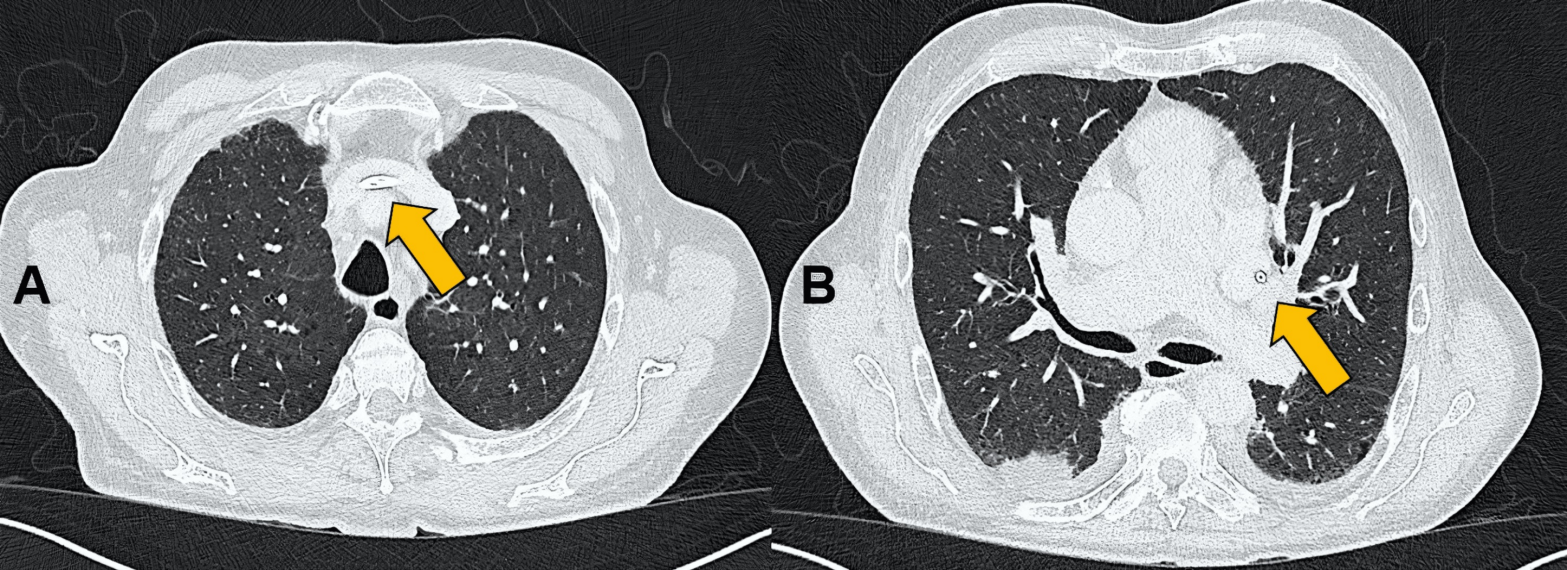
CT angiographic confirmation of an isolated persistent left superior vena cava (PLSVC) with absence of the right superior vena cava. (A) Axial contrast-enhanced CT angiography at the upper thoracic level demonstrating complete absence of a right-sided superior vena cava (orange arrow). (B) More caudal section showing a large-caliber PLSVC descending along the left mediastinum and draining into a markedly dilated coronary sinus posterior to the left atrium (orange arrow).

A catheter was inserted to a depth of 23 cm, with its tip located in the anomalous vessel. Although initial aspiration and injection were unremarkable, thoracic CT angiography was performed immediately thereafter to clarify the atypical venous course observed fluoroscopically. The CT confirmed a PLSVC draining into a markedly dilated coronary sinus with complete absence of a right-sided superior vena cava (Figure 2).

The azygos system drains directly into the PLSVC. Additional findings included cardiomegaly with biatrial enlargement, a left atrial appendage thrombus, and bilateral pleural effusions.

**Figure 3:**
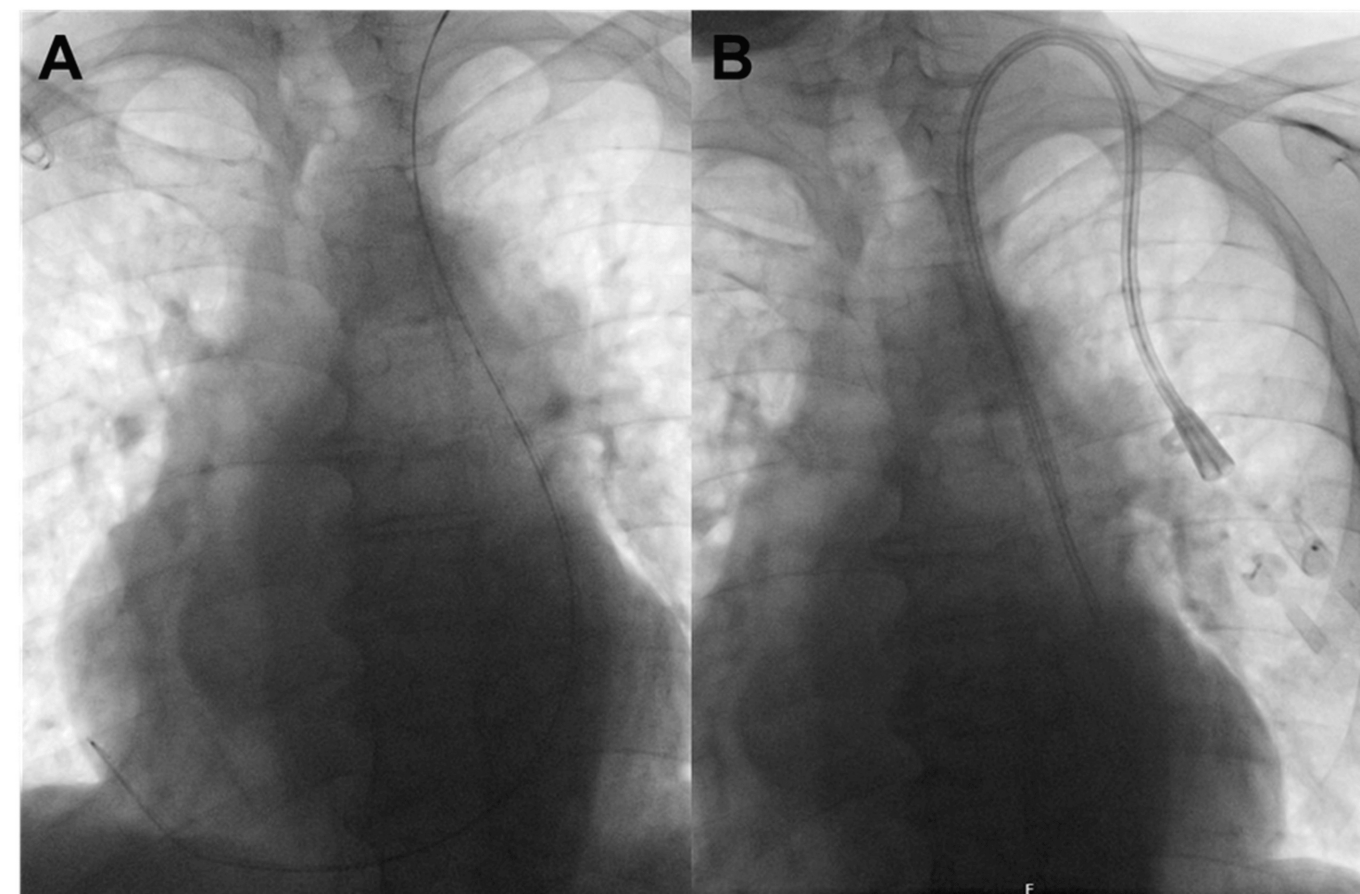
Fluoroscopic confirmation of successful catheter reimplantation via the left internal jugular vein in an isolated persistent left superior vena cava (PLSVC). (A) Fluoroscopic image during reimplantation via the left internal jugular vein, demonstrating deeper intrathoracic advancement of a dual-lumen tunneled hemodialysis (Demers) catheter with a left-sided mediastinal course consistent with passage through a PLSVC. (B) Post-procedural chest radiograph demonstrating a straight left mediastinal trajectory and correct catheter positioning within the PLSVC, ensuring sufficient intrathoracic depth.

The right-sided catheter was removed the following day without complication. On October 16, 2025, a new tunneled catheter was inserted via the left internal jugular vein under ultrasound and fluoroscopic guidance, with a tip depth of 27 cm to ensure proper positioning (Figure 3A).

During advancement, transient atrial ectopy occurred, and final placement within the right atrium was avoided. The catheter tip was positioned deep within the large-caliber PLSVC with unobstructed aspiration and injection. The lumens were locked with citrate solution. Post-procedural chest radiography demonstrated a straight left mediastinal course consistent with correct positioning in the PLSVC (Figure 3B).

The subsequent dialysis sessions showed stable blood flow rates of 300-350 mL/minute and adequate clearance. No further arrhythmias or complications occurred. The patient’s clinical condition improved markedly with effective ultrafiltration, and he was discharged in stable condition after five days.

This case illustrates how an unrecognized PLSVC, particularly in the absence of a right SVC, may result in ineffective hemodialysis and emphasizes the importance of careful imaging interpretation during central venous catheter placement.

## Discussion

PLSVC is a well-recognized venous anomaly, but it is usually clinically relevant only in specific procedural contexts. The present case illustrates a particularly uncommon variant - PLSVC in the absence of a right SVC - discovered incidentally during hemodialysis catheter placement.

This configuration can complicate central venous access and represents a potential pitfall for interventional nephrologists, radiologists, and cardiologists. Beyond access considerations, isolated or dual-SVC anatomy can be pertinent to rhythm management. In cohorts of patients undergoing atrial fibrillation (AF) ablation, PLSVC has been identified in ~0.9% and acted as a trigger or driver of AF in a substantial subset, underscoring the value of targeted electrophysiological assessment when present [12].

Embryologically, PLSVC results from persistence of the left anterior cardinal vein, while absence of the right SVC - as observed in our patient - occurs in fewer than 0.1% of individuals [13]. This configuration reflects persistence of the left anterior cardinal vein with regression of the right-sided component, yielding an isolated left-sided caval system [14]. The cardinal venous network, formed in the fourth week of development, consists of paired anterior and posterior veins draining into the sinus venosus horns; deviations in formation or regression of these channels explain most variants of the superior caval system [15-17].

In our patient, digital subtraction angiography during initial guidewire advancement revealed an anomalous leftward course, prompting immediate CT angiography before catheter use. Cross-sectional imaging confirmed a large-caliber PLSVC draining into a markedly dilated coronary sinus and complete absence of a right-sided SVC, explaining the unexpected guidewire trajectory. Echocardiography can suggest a dilated coronary sinus, but is often limited for comprehensive characterization due to acoustic shadowing from bone and lung. By contrast, CT and MR venography enable direct visualization of the venous pathway; CT venography optimally enhances the superior vena cava approximately 60-75 seconds after peripheral contrast injection [18]. Multiplanar and volume-rendered reconstructions further support procedural planning by clarifying the access route and the appropriate catheter length.

These imaging findings guided successful reimplantation via the left internal jugular vein with deeper intrathoracic positioning of the cuffed, dual-lumen catheter, which restored adequate dialysis flow. The observed coronary-sinus enlargement aligns with its embryologic derivation from the left sinus venosus horn and the left common cardinal vein [15]. A left atrial appendage thrombus was also present.

While our patient’s venous anomaly drained into the right atrium, preventing a right-to-left shunt, left atrial drainage or unroofed coronary-sinus variants have been described as risk factors for paradoxical or septic emboli [19]. The atrial thrombus here was most likely attributable to chronic AF and heart failure rather than the venous anomaly itself; nonetheless, this constellation emphasizes caution when manipulating catheters within a dilated coronary sinus in patients with intracardiac thrombi.

Most reported PLSVCs identified during central venous or device implantation procedures coexist with a normally developed right SVC and drain into the right atrium via the coronary sinus, rendering the anomaly usually hemodynamically insignificant. Nevertheless, as several reports note, even such anatomically benign variants can complicate guidewire or lead navigation and occasionally provoke transient arrhythmias during manipulation [9-11].

By contrast, isolated PLSVCs - particularly those without a right-sided counterpart or with atypical drainage - pose distinctive procedural challenges due to altered venous return, coronary-sinus dilation, and limited options for optimal catheter positioning. Awareness of this variant during fluoroscopic or angiographic guidance is essential. In patients with end-stage kidney disease, especially those with atypical thoracic anatomy or prior catheterization difficulties, preprocedural venous imaging should be considered to prevent catheter malfunction, repeated interventions, or embolic complications.

In practice, several lessons emerge from this case: recognition of an atypical leftward guidewire course should prompt immediate imaging to delineate venous anatomy before catheter use; isolated PLSVC can substantially alter venous return and complicate catheter placement; CT angiography offers rapid anatomic clarification with actionable procedural guidance; and management is most effective when coordinated through a multidisciplinary approach involving nephrology, radiology, and cardiology.

## Conclusions

This case illustrates that a persistent left superior vena cava without a right-sided counterpart, although rare, can materially affect central venous access and hemodialysis planning. Recognition of the anomaly at the time of guidewire advancement is critical: an atypical leftward course should prompt immediate fluoroscopy and, when needed, cross-sectional imaging before catheter use. Early identification enables appropriate access selection and positioning and helps prevent malposition, inadequate flow, or procedure-related arrhythmia. A multidisciplinary approach involving nephrology, interventional radiology, and cardiology optimizes diagnosis and management. Awareness of this anatomical variant can prevent complications and improve vascular access outcomes in patients requiring dialysis. A high index of suspicion for venous anomalies should always be maintained when guidewire behavior deviates from the expected course during central venous access.
